# Integrating proteomics with electrochemistry for identifying kinase biomarkers†
†Electronic supplementary information (ESI) available. See DOI: 10.1039/c5sc00560d



**DOI:** 10.1039/c5sc00560d

**Published:** 2015-05-22

**Authors:** Einav Amit, Rofeamor Obena, Yi-Ting Wang, Roman Zhuravel, Aaron James F. Reyes, Shir Elbaz, Dvir Rotem, Danny Porath, Assaf Friedler, Yu-Ju Chen, Shlomo Yitzchaik

**Affiliations:** a Institute of Chemistry and the Center for Nanoscience and Nanotechnology , the Hebrew University of Jerusalem , Safra Campus, Givat Ram , Jerusalem 91904 , Israel . Email: Shlomo.yitzchaik@mail.huji.ac.il ; Email: assaf.friedler@mail.huji.ac.il; b Institute of Chemistry , Academia Sinica , Taipei , Taiwan . Email: yujuchen@gate.sinica.edu.tw; c Molecular Science and Technology Program , Taiwan International Graduate Program , Taipei , Taiwan; d Department of Chemistry , National Tsing Hua University , Hsinchu , Taiwan

## Abstract

We present an integrated approach for highly sensitive identification and validation of substrate-specific kinases as cancer biomarkers. Our approach combines phosphoproteomics for high throughput cancer-related biomarker discovery from patient tissues and an impedimetric kinase activity biosensor for sensitive validation. Using non-small-cell lung cancer (NSCLC) as a proof-of-concept study, label-free quantitative phosphoproteomic analysis of a pair of cancerous and its adjacent normal tissues revealed 198 phosphoproteins that are over-phosphorylated in NSCLC. Among the differentially regulated phosphorylation sites, the most significant alteration was in residue S165 in the Hepatoma Derived Growth Factor (HDGF) protein. Hence, HDGF was selected as a model system for the electrochemical studies. Further motif-based analysis of this altered phosphorylation site revealed that extracellular-signal-regulated kinase 1/2 (ERK1/2) are most likely to be the corresponding kinases. For validation of the kinase–substrate pair, densely packed peptide monolayers corresponding to the HDGF phosphorylation site were coupled to a gold electrode. Phosphorylation of the monolayer by ERK2 and dephosphorylation by alkaline phosphatase (AP) were detected by electrochemical impedance spectroscopy (EIS) and surface roughness analysis. Compared to other methods for quantification of kinase concentration, this label-free electrochemical assay offers the advantages of ultra-sensitivity as well as higher specificity for the detection of cancer-related kinase–substrate pair. With implementation of multiple kinase–substrate biomarker pairs, we expect this integrated approach to become a high throughput platform for discovery and validation of phosphorylation-mediated biomarkers.

## Abstract 2

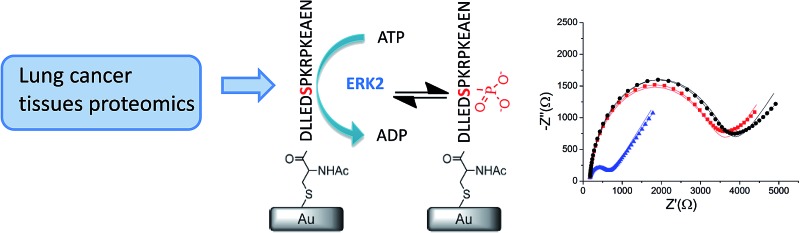
We present an integrated approach for highly sensitive identification and validation of substrate-specific kinases as cancer biomarkers.

## Introduction

The human kinome contains over 500 identified kinases^1^ that create a vast network of kinase cascades.^2^ Kinases play central roles in cell signaling and abnormal kinase-mediated phosphorylation is strongly linked to cancer.^3–5^ Therefore, kinases are important for cancer therapy at two levels: on one hand, they are promising drug targets and various kinase inhibitors are developed as anti-cancer leads.^6^ On the other hand, kinases are important cancer biomarkers that enable early detection of cancer.^7–9^ However, a systematic study on the regulation of kinase activation and association with cancer at the proteomic network level *in vivo* has not been performed. Developing kinase-based diagnostic tools for cancer involves the identification of the target kinase whose expression is altered in cancer, followed by the development of sensitive methods for detecting the activity of this specific kinase.^10–12^ The aberrant kinase could ubiquitously affect the signal transduction networks across multiple pathways. A kinase detection method capable of detecting its phenotype-dependent substrates may provide better specificity to monitor a disease related to a specific pathway. Due to their medical and biological significance, many methods for the detection of kinase activity have been previously demonstrated. These include radiometric assays,^13^ spectroscopic assays^11,12,14,15^ and electrochemical methods. Electrochemical methods have the advantages of avoiding work with radioactive materials, higher detection sensitivities and consumption of lower amounts of analyte without the need for amplification systems.^16,17^ Diverse applications of electrochemical methods for detecting kinase activity were reported. These include the use of ferrocene labeled ATP^10^ or detection with antiferrocene antibodies in an immunoarray format.^18^ In label-free methods, the detection is based on repulsion between the negatively charged phosphate groups and negatively charged redox species in the solution that is translated in EIS measurements to increase in the resistance to charge transfer (*R*_CT_).^19^ Alternatively, there is attraction of a cationic redox species to the phosphate group on the peptide that is translated in cyclic voltammetry (CV) measurements to increase in current.^16,20^ We have previously reported a label-free electrochemical kinase detection method, which is based on a peptide monolayer that changes its conformation upon phosphorylation from a densely packed monolayer into a disordered one, causing a sharp drop in *R*_CT_. This approach has a built-in amplification mechanism, which is based on a reversible order–disorder transition that the peptide monolayer undergoes due to its phosphorylation. This order–disorder transition changes the electronic properties of the layer from a good ionic insulator to an ion-permeable peptide layer and does not rely only on ionic repulsion/attraction from the phosphorylated peptide layer.^20^

Here we report an integrated platform combining quantitative tissue phosphoproteomics for kinase–substrate biomarker discovery and electrochemical kinase detection method for ultra-sensitive kinase–substrate biomarker validation. This can be used as a step towards individualized cancer diagnostics. Phosphoproteomic approaches have demonstrated effective identification of the abnormal protein kinase activity involved in cancer.^21,22^ Using NSCLC as a proof-of-concept study, label-free quantitative phosphoproteomic analysis of a pair of cancerous and its adjacent normal tissues revealed site-specific elevated phosphorylated proteins/peptides in NSCLC. Through sequence motif-analysis and literature mining among the 198 most dramatically up-regulated phosphopeptides, HDGF was chosen as a model protein for the electrochemical studies. A peptide derived from the HDGF phosphorylation site was coupled to a gold electrode and electrochemical impedance spectroscopy was utilized to probe its phosphorylation by ERK2 and dephosphorylation by AP. The unification of proteomics and electrochemistry enabled us both to identify the cancer-related target sequence of the kinase and to detect the kinase levels at low concentrations. The developed method is general and could be applied to large-scale biomarker discovery and validation for other diseases such as other cancer types or neurodegenerative diseases.

## Results

### Identification of over-activated phosphoproteins in NSCLC by phosphoproteomics

**Fig. 1:**
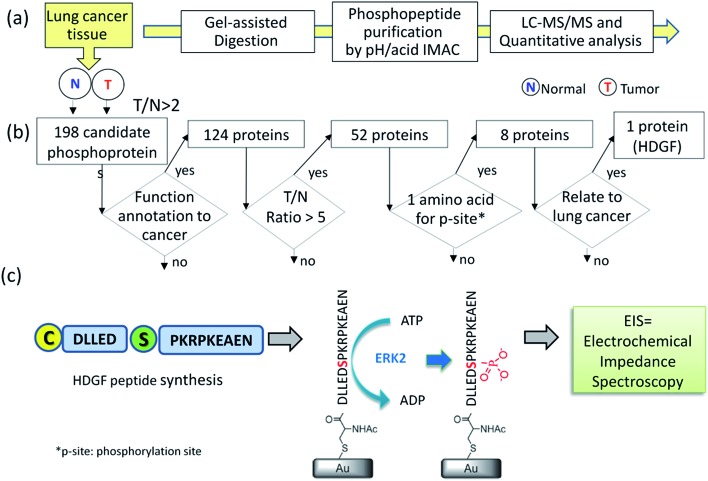
A strategy for selecting the target HDGF–kinase pair assay. (a) Identification of HDGF by quantitative phosphoproteomics analysis of 66 year-old male patient tissues. A paired tissue analysis of the tumor tissue and the adjacent normal tissue samples were subjected to gel-assisted digestion, purification for phosphopeptides by automated pH/acid-controlled IMAC, and triplicate LC-MS/MS analysis. Bioinformatics analysis by IDEAL-Q software was used to process the quantitation result for peptide–kinase pair selection; (b) a decision tree for selecting the target peptide for synthesis. *T*/*N* = amount of phosphorylation in tumor tissue/amount of phosphorylation in normal tissue; (c) design, synthesis, and electrochemical detection of kinase-promoted phosphorylation in HDGF peptide.

To identify substrate/kinase pair that are over-activated in lung cancer and potentially serve as biomarkers, we first searched for phosphoproteins that are over-phosphorylated in lung cancer tissue in comparison with its corresponding adjacent normal tissue from the same patient. The altered phosphorylation level may represent abnormal kinase activity in this specific cancer. A label-free quantification method (Fig. 1a) integrating a gel-assisted digestion and pH/acid-controlled IMAC protocol was used for phosphoproteomic analysis of a pair of cancerous and adjacent tissues from patients with NSCLC.^23–25^ The phosphopeptides enriched from the IMAC were analyzed by LC-MS/MS (Triple-TOF 5600) for phosphoproteomic analysis. A total of 1451 phosphopeptides (derived from 540 phosphoproteins) were confidently identified by the Mascot search engine and quantified by the IDEAL-Q software.^26^ To identify kinase/substrate pairs that are over-activated and can serve as biomarkers involved in NSCLC, the extent of over-phosphorylation of every phosphopeptide in the tumor tissue was quantitatively compared by defining: *T*/*N* ratio = amount of phosphorylation in tumor tissue/amount of phosphorylation in adjacent normal tissue. The specific target phosphoprotein was selected by a series of steps (Fig. 1b). Several bioinformatics tools together with literature mining were used to search for the target kinase–substrate pairs. First, we selected phosphopeptides that had a *T*/*N* ratio >2 since we considered those to be up-regulated in the tumor tissue with statistical significance. The quantification results revealed >2-fold up-regulation of 386 phosphopeptides from 198 phosphoproteins (see details in ESI Table 1†). Among them, 124 originate from proteins reported to be related to cancer by Metacore™ analysis. Of these peptides, 85 phosphopeptides derived from 52 phosphoproteins, underwent significantly increased phosphorylation in the tumor tissue, *i.e.* ≥5-fold *T*/*N* ratio.

As an initial model system, we selected phosphopeptides that have only one possible phosphorylation site per peptide. Of the eight remaining proteins (Table 1), only HDGF is overexpressed (6-fold) in lung cancer, and was therefore selected.^27,28^ The HDGF peptide sequence is ^160^DLLEDpSPKRPKEAEN^174^, with a phosphorylation site at position S165 (Table 1). We also evaluated whether HDGF was also overexpressed at the protein level. The results revealed that the protein level of HDGF also show overexpression in tumor tissue compared to adjacent normal tissue from the same patient (ESI Fig. 1†). Therefore, detection of its kinase activity by the developed kinase assay is very important to understand the phosphorylation alteration of HDGF protein.

**Table 1:** A list of phosphoproteins and their phosphorylation sites that show increased levels in lung cancer tissue compared to adjacent normal tissue^*a*^

Acc. no	Gene name	Protein name	*m*/*z*	Charge	Modification site	303T/303N
Q13586	STIM1	Stromal interaction molecule 1	469.21	3	AEQS[Phospho (ST)]LHDLQER	12
**P51858**	**HDGF**	**Hepatoma-derived growth factor**	**753.38**	**2**	**AGDLLEDS[Phospho (ST)]PKRPK**	**6**
P00558	PGK1	Phosphoglycerate kinase 1	616.99	3	ALES[Phospho (ST)]PERPFLAILGGAK	25
P13796	LCP1	Plastin-2	615.76	2	EGES[Phospho (ST)]LEDLMK	11
P49736	MCM2	DNA replication licensing factor MCM2	987.41	2	GLLYDS[Phospho (ST)]DEEDEERPAR	12
Q9UKM9	RALY	RNA-binding protein Raly	579.31	2	GRLS[Phospho (ST)]PVPVPR	9
Q14004	CDC2L5	Cell division cycle 2-like protein kinase 5	505.93	3	ILELT[Phospho (ST)PEPDRPR	7
P37802	TAGLN2	Transgelin-2	680.28	2	NFS[Phospho (ST)]DNQLQEGK	7

^*a*^The *T*/*N* ratio for each peptide refers to the amount of phosphorylation in tumor tissue/amount of phosphorylation in adjacent normal tissue. The HDGF peptide is highlighted.

### Identification of the target kinase

**Fig. 2:**
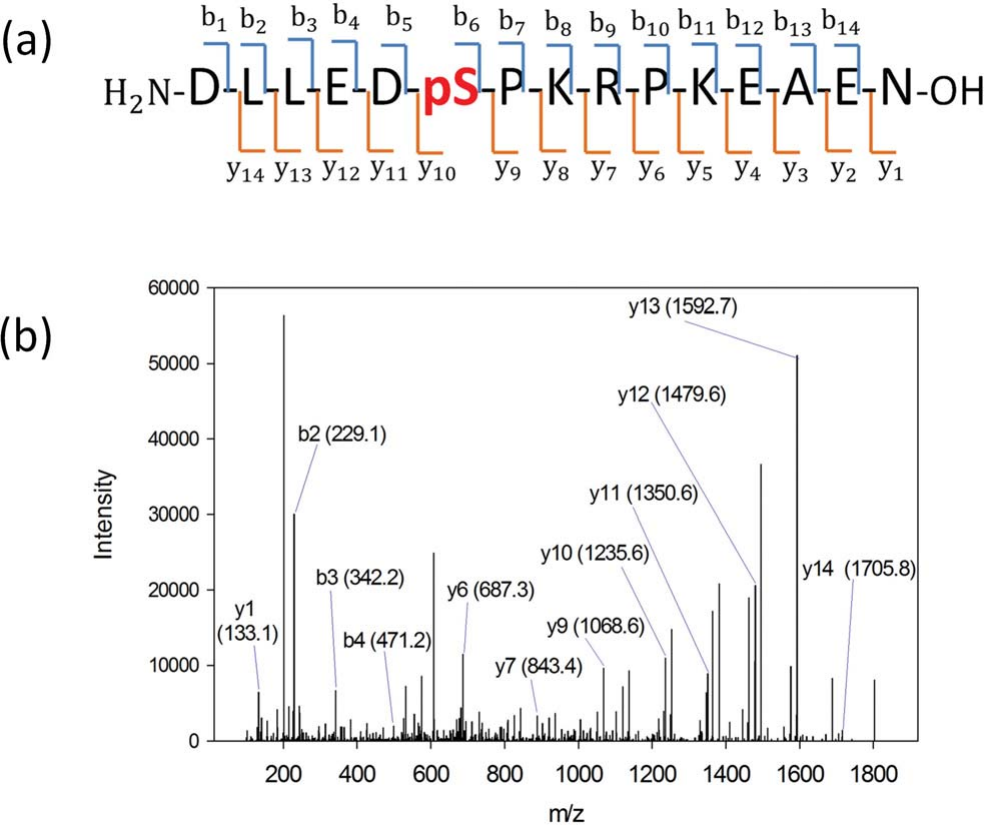
(a) HDGF phosphopeptide sequence with b- and y-ion assignments observed in MS/MS spectrum. A series of y-ions (y_10_–y_14_) containing the phosphate group. (b) The MS/MS spectrum of the HDGF phosphopeptide. The arrows indicate the ions containing the phosphate group. The observed *m*/*z* is 607.62 with charge of 3+ which corresponds to the HDGF phosphopeptide peak.

We searched for the specific kinase that phosphorylates S165 in HDGF *via* on-line system biology resources, including PhosphoSitePlus® and the Human Protein Reference Database (HPRD) website-based software.^29–31^ ERK1/2 were shown to be potential kinases that are able to phosphorylate HDGF on serine 165 (Table 1).^25,32^ To verify that these kinases indeed phosphorylate the HDGF 160–174 peptide, an *in vitro* kinase assay was performed at both peptide and protein levels. The products of the peptide phosphorylation by ERK2 were analysed by LC-MS/MS and the phosphopeptide signal was observed at the expected mass/charge (*m*/*z*) value (607.62 for charge of 3^+^). The serial of y-fragment ions (y_10_–y_14_) retaining the phosphate group in the MS/MS spectrum shown in Fig. 2b confirmed that S165 is the phosphorylation site by ERK2. We also performed the same *in vitro* kinase reaction at the protein level. The ERK kinase reaction for the dephosphorylated HDGF protein showed the same phosphorylation site at serine 165 (see details in ESI Fig. 2†).

### Assembly of the HDGF peptide on gold electrodes

**Fig. 3:**
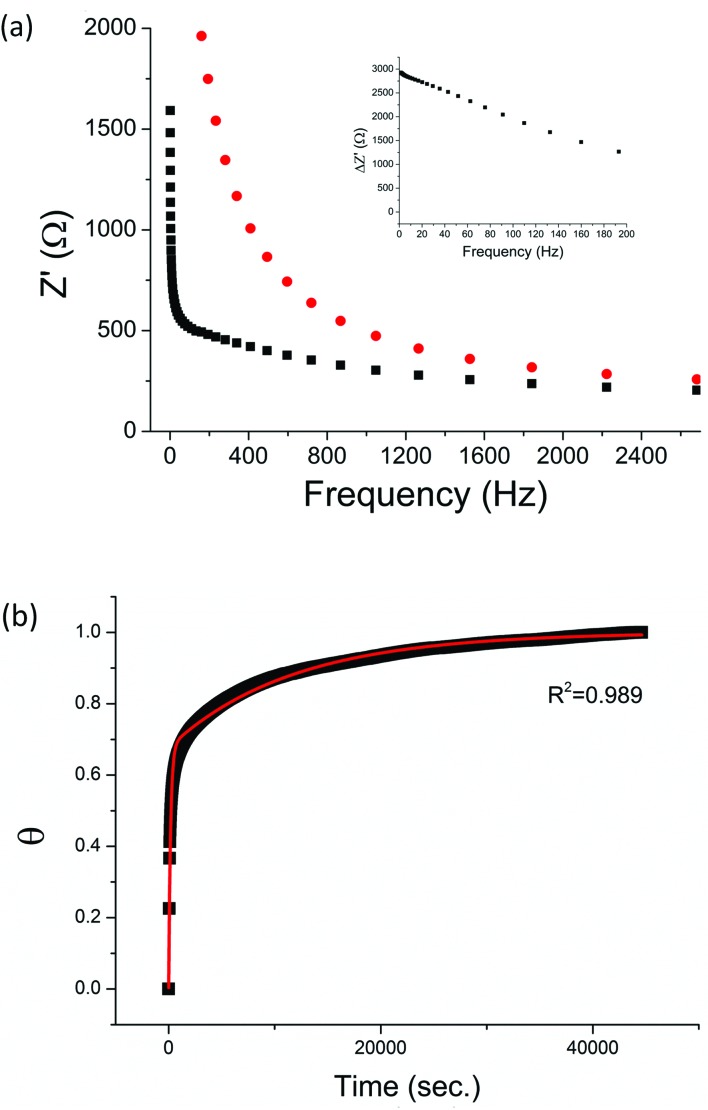
(a) Bode plots of a clean gold electrode (black square) and the same electrode after peptide adsorption overnight (red circle). Inset: the difference in the real impedance (*Z*′) between the peptide coated electrode and the clean gold electrode (Δ*Z*′). (b) Adsorption of HDGF 160–174 onto a clean gold electrode. Relative surface coverage (*θ*) was plotted against time (■). The curve was fit to a second order reaction kinetic equation (red line).

The non-phosphorylated form of the selected HDGF peptide was synthesized with an N-terminal cysteine added for binding to gold electrodes (Fig. 1c, see above) and its electrochemical response to phosphorylation was examined. To determine the optimal assembly time of the peptide on the electrode, we performed *in situ* kinetic impedimetric measurements (Fig. 3). The frequency for the kinetic measurements was selected to be 100 Hz, because in this frequency the impedance of the peptide monolayer was significantly higher than that of the clean gold (see Fig. 3a, inset) and not too low to be diffusion controlled. The *in situ* adsorption experiment (Fig. 3b) showed that the real impedance (*Z*′) increased over time and was at a plateau after 16 hours. We concluded that 16 hours of incubation is the optimal time for the adsorption of the peptide. The data was transferred to surface coverage (*θ*) using the expression:
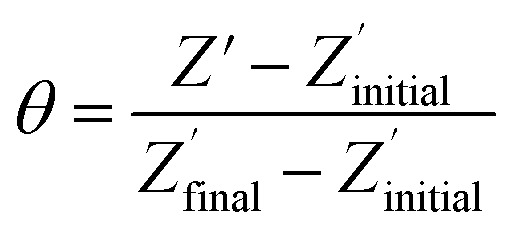
with *Z*′ being the real impedance at each point, *Z*′initial – the value of the clean gold and Z′final – the final value at the plateau. The adsorption isotherm was fit to the equation:^33,34^(1 – *θ*) = *α*e^–*k*_1_*t*[*C*]^ + (1 – *α*)e^–*k*_2_*t*[*C*]^where *θ* is the fractional surface coverage, *k*_1_ and *k*_2_ are the rate constants that were found to be *k*_1_ = 55.5 ± 0.6 M^–1^ s^–1^ and *k*_2_ = 0.858 ± 0.004 M^–1^ s^–1^, *C* – the initial concentration of the peptide solution (0.1 mM). The value of the coefficient *α* is 0.677 ± 0.001.

**Fig. 4:**
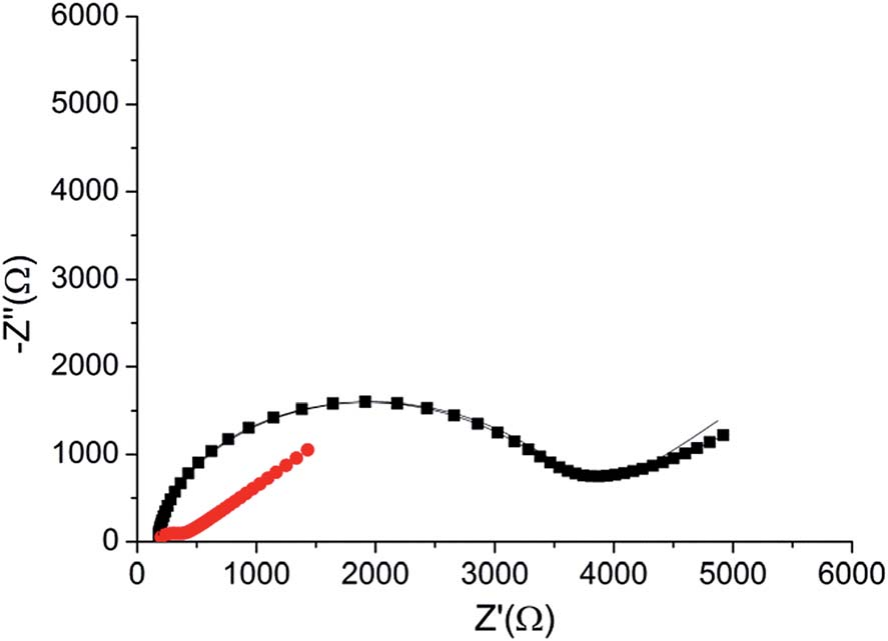
Nyquist plot of the HDGF 160–174 monolayer on the electrode (black square), compared with that of a bare gold electrode (red circle). Lines – fits to the equivalent circuit [*R*(*C*[*RW*])] (see, Fig. 5 inset). The monolayer was created by incubating a weakly basic 0.1 mM solution of the peptide on a clean gold electrode for 16 hours. EIS spectrum was obtained at a frequency range of 0.1 Hz–10 kHz with amplitude of 10 mV, at the formal potential of the redox couple *vs.* Ag/AgCl electrode (0.17 V).

The peptide formed a dense monolayer, as observed by the high *R*_CT_ of the electrode after incubation with the peptide (3400 Ω) compared with the impedance of the bare gold electrode (380 Ω) (Fig. 4). The increase in *R*_CT_ is caused by a layer blocking the electron transfer from the redox species in the solution to the electrode.

**Fig. 5:**
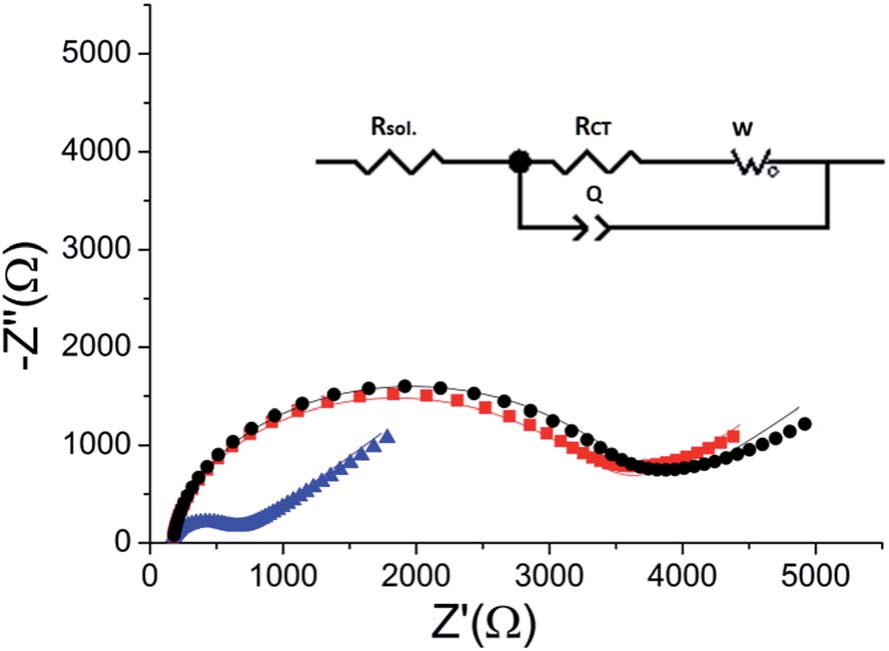
Reversibility of the phosphorylation – dephosphorylation process. Black (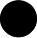) – the *R*_CT_ of the peptide monolayer (*R*_CT_ = 3400 Ω). Blue (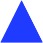) – the *R*_CT_ of the monolayer following phosphorylation with ERK2 (*R*_CT_ = 474 Ω). Red (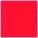) – the impedance of the monolayer after dephosphorylation with AP (*R*_CT_ = 3250 Ω). Lines – fits to the equivalent circuit (inset).

### ERK2-mediated phosphorylation of the HDGF peptide

ERK2-mediated phosphorylation of the peptide monolayer resulted in a decrease in *R*_CT_, showing that phosphorylation indeed took place (Fig. 5). The results were fit to a Randles type circuit [*R*_sol._(*Q*[*R*_CT_ *W*])](Fig. 5 inset). The results are summarized in Table 2. The parameters are the solution resistance, *R*_sol._, the resistance to charge transfer, *R*_CT_, *Q* the constant phase element, and its exponent, *α*.
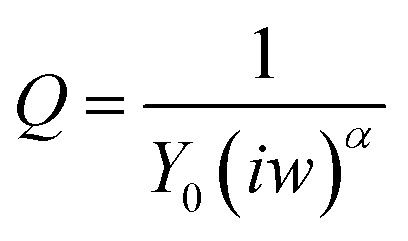


**Table 2:** The fit values of the different circuit elements for the HDGF-derived peptide monolayer before phosphorylation, after phosphorylation with ERK2, and after dephosphorylation with AP

	*R*_sol._ (Ω)	*α*	*Q* (nF)	*R*_CT_ (Ω)	*W* (μF)
Peptide	170 ± 1	0.940 ± 0.003	0.42 ± 0.01	3400 ± 20	0.211 ± 0.004
ERK2	161 ± 3	0.90 ± 0.01	0.83 ± 0.09	474.1 ± 8	0.250 ± 0.006
AP	168 ± 3	0.940 ± 0.01	0.64 ± 0.09	3250 ± 8	0.245 ± 0.001

Following incubation with ERK2, the *R*_CT_ decreased by 86% to 474 Ω. The reversibility of the phosphorylation reaction was examined by performing dephosphorylation with Alkaline Phosphatase (AP). Following dephosphorylation, the *R*_CT_ increased back to 3250 Ω – almost to the starting value. A reversible change is evident also in *α*, the exponent, changing after phosphorylation from 0.94 to 0.90 and changing back to 0.94 after dephosphorylation.

### Selectivity of the assay – phosphorylation with calmodulin kinase 2 (CaMK2)

**Fig. 6:**
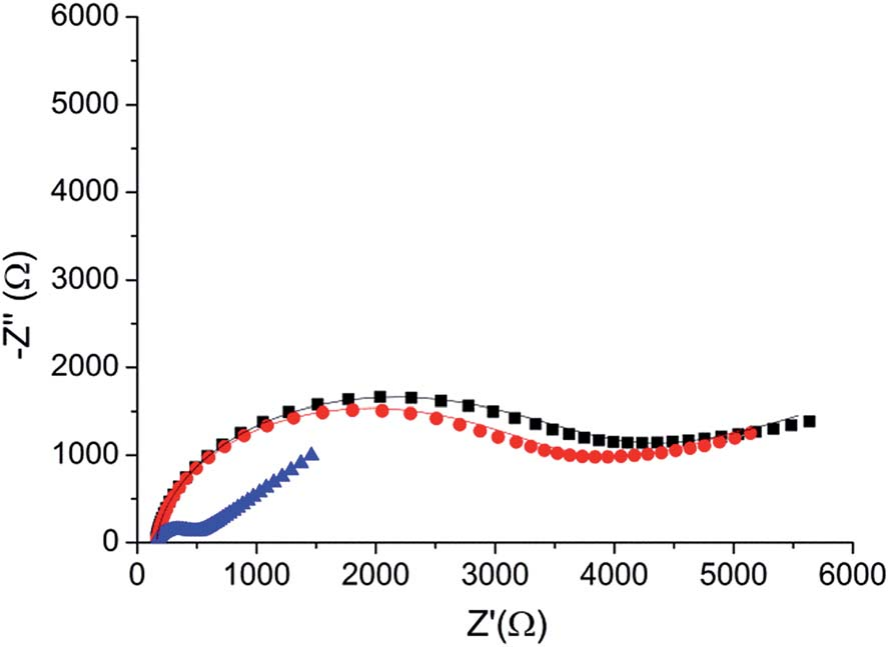
A phosphorylation process with CaMK2. Black (■) – the *R*_CT_ of the peptide monolayer (*R*_CT_ = 4080 Ω). Red () – the *R*_CT_ of the monolayer following phosphorylation with CaMK2 (*R*_CT_ = 3600 Ω). Blue (▲) – the *R*_CT_ of the monolayer after phosphorylation with ERK2 (*R*_CT_ = 390 Ω). Lines – fits to the equivalent circuit.

To verify the selectivity of the substrate-dependent kinase assay, the same process of phosphorylation and dephosphorylation on HDGF 160–174-covered electrodes was tested with CaMK2, which is not known to phosphorylate the HDGF 160–174 sequence (Fig. 6). After incubation with CaMK2, the *R*_CT_ decreased by only 12% from 4080 Ω to 3600 Ω. This shows that CaMK2 has much lower activity towards HDGF 160–174 and thus demonstrates the good selectivity of our assay.

### Monitoring the phosphorylation by monolayer roughness changes using atomic force microcopy (AFM)

**Fig. 7:**
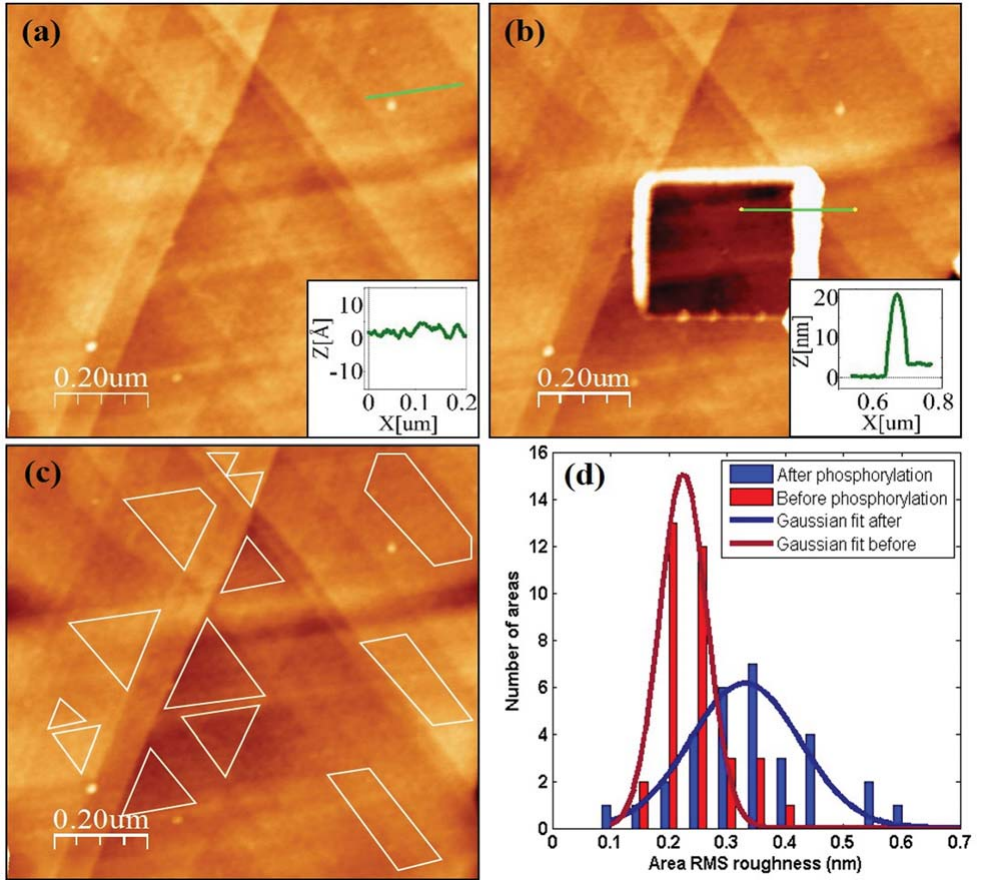
(a) HDGF 160–174 layer on top of annealed gold substrate. The layer looks very smooth and uniform. The inset is a cross section along the green line (on single gold terrace) with RMS roughness of ∼0.3 nm. (b) Square scratch in the peptide layer. The inset is a profile along the green line, showing a ∼2 nm height difference between scratched and non-scratched areas. (c) An example of several small flat areas for roughness analysis on a single gold terrace. (d) Roughness distribution of ∼60 areas at macroscopically distant scans before and after phosphorylation.

A direct and independent method to verify that the phosphorylation affects the monolayer was implemented by monitoring changes of the monolayer roughness before and after phosphorylation. For this purpose we used an annealed gold substrate that is composed of large single crystal grains (up to several μm^2^). The orientation of most facets on the grains reveals the (111) plane of the fcc structure and have a triangular shape.^35^ Each triangle is an atomically flat surface. AFM characterization of annealed gold covered with the HDGF 160–174 peptide revealed a very smooth and homogeneous surface (Fig. 7a), generally resembling the appearance of the annealed gold surface. Square scratch of the surface by the AFM tip proved that there is a relatively soft, ∼2 nm thick, layer on top of the annealed gold (Fig. 7b), indicating very dense and seemingly homogeneous peptide coverage.

Root mean squared (RMS) roughness was calculated on small areas, 10 000–100 000 nm^2^, with a single “gold terrace” (examples in Fig. 7c). The high sampling resolution enabled detailed height distribution analysis even on small areas. At these areas the substrate is atomically flat with RMS roughness of less than 0.2 nm. Therefore, the measured roughness originated mainly from the covering peptide layer. Sixty such areas were examined in different scans at locations with distance of few mm between them, before and after phosphorylation. Roughness distribution of these areas is shown in Fig. 7d. The phosphorylation widens the roughness distribution and shifts its peak from ∼0.2 (red) to higher values of ∼0.35 (blue). This means that the surface roughness becomes higher and less homogeneous after phosphorylation. This could be either due to small defects in the dense arrangement of peptides on the gold surface, *e.g.*, pinholes, or due to disturbance of the surface packing upon phosphorylation, or both. In either case this result is a clear indication for a change in the monolayer packing due to phosphorylation.

## Discussion

In this work we present a novel approach for kinase related cancer research. We show that discovery and detection of over-phosphorylated proteins and the relative kinases can be done in a relatively simple manner, which is very versatile. Methods for detection of phosphorylated proteins in tissues had been suggested before^36–38^ and so were detection methods for specific kinases.^11,14,16,17^ We combine the two aspects and show a direct path from the tissue level to the kinase detection stage. Specifically, we show that the over-phosphorylation level of proteins in tumour samples can be determined by proteomic tools. The identification of over-phosphorylated residues in a target protein leads to kinase identification and the use of a peptide containing the phosphorylation site in an electrochemical assay provides a powerful tool for kinase detection. Our detection method is unique, because it is label-free and very simple – the biosensor is composed of only the peptide and a simple gold macro electrode. This is unlike other electrochemical kinase detection methods that utilize a variety of labels and amplification methods.^39–42^

### Tissue phosphoproteomics identified over-activated kinase–substrate pairs in NSCLC

Protein kinases are highly attractive targets for therapeutic intervention. Therefore, identification of their pathway-associated phosphorylation substrates may generate biomarkers to aid either the disease detection or the drug development process. The selection of the kinase/substrate target is the most crucial aspect of drug target identification. Proteomics technology is a powerful tool for discovering aberrant protein expression and post-translational modification associated with disease. The polyMAC has been applied to tissue phosphoproteomics in transgenic mouse model of HER2 positive breast cancer and identified potential therapeutic targets.^36^ Phosphoproteomics has also been applied to directly identify kinase inhibitor targets in cancer cell lines and mouse xenografts.^43^ Though these studies show the promise of phosphoproteomics to provide new targets for designing anticancer therapies, most current approaches are not accessible to individualized human tissue analysis yet. This advantage of our approach is critical since it enables to reflect the human microenvironment as well as the difference between individual patients.

To our knowledge, our study is the first report of an individualized phosphoproteomic analysis on a pair of cancerous and adjacent normal tissues from the same patient. By our sensitive label-free quantitative phosphoproteomic strategy, the results revealed identification of over one thousand phosphorylation substrates in the NSCLC tumour tissue. Hundreds of abnormally activated phosphorylation substrates enriched in human lung cancer tissue served as starting point to search for their putative kinases by motif-based informatics analysis. By comparing to adjacent normal tissues, we could largely reduce the individual difference between samples. Through the logical dissection of the phosphoproteomics data by bioinformatics tools, the HDGF peptide and the corresponding ERK2 kinase were studied by the electrochemical impedance spectroscopy method. In addition to HDGF, dozens of up-regulated phosphoproteins identified by our platform had dramatic change in expression in the tumour tissue compared to the normal tissue and also passed our criteria since their function is related to cancer. With the added advantage of multiplexed phosphopeptide enrichment by automatic IMAC protocol, the phosphoproteomic method described herein is an efficient strategy for identifying the full panel of substrate–kinase biomarker pairs and can be applied in a large cohort of clinical samples.

### Substrate-dependent kinase monitoring by an electrochemical assay

To provide a facile and sensitive assay to monitor the substrate-dependent kinase activity, we have devised an electrochemical assay using peptide-coated gold electrodes. The peptide assembly on the electrode is carried out in two steps – a fast adsorption process, beginning right at the moment of peptide injection, followed by a second, slower process. The rate constants show that the first adsorption process lasts until about two thirds of the adsorption sites are bound. Then the second process, which is likely to be an interaction between the peptides in the solution and the adsorbed peptide layers, takes place and slows down the adsorption of more peptides onto the surface. We interpret this barrier in terms of increasing peptide–peptide electrostatic and steric repulsion with increasing coverage. After the adsorption, the resulting monolayer was dense, as observed by the high *R*_CT_ values obtained compared with other studies.^10,16^ Previously, high initial *R*_CT_ gave us better sensitivity in this system. The introduction of the negatively charged phosphate groups into a denser monolayer causes a more prominent change, resulting in a larger change in the *R*_CT_ following phosphorylation. This is evident in Fig. 5, where there is a six – fold decrease in the *R*_CT_ after phosphorylation.

A decrease in *R*_CT_ can be caused by many reasons, among them detachment of the peptides from the monolayer. We showed that the process is reversible, and harmless to the monolayer, by performing the dephosphorylation reaction with AP. AP removes the phosphate groups from the peptide, resulting in free hydroxyl groups. We observed that following dephosphorylation, the *R*_CT_ of the peptide monolayer increased again, practically to the same starting value as of the native peptide. This reversible change may also indicate a change in the properties of the monolayer. Upon phosphorylation, the order of the monolayer is disrupted and more water molecules penetrate into the monolayer, facilitating the passage of the redox species and making it more conductive. The larger hydration layer of the phosphate group relative to the hydroxyl group most likely also influences the dielectric properties of the monolayer and therefore the diffuse layer of the electrode.

Fitting the plots to the equivalent circuit shown in Fig. 5 provided us with additional information about the changes on the surface. The reversible change in *α*, the exponent of the constant phase element, indicates a change in the homogeneity of the surface.^44,45^ The values of the parameter *α* are usually between 0–1, 1 indicating a perfect capacitor, and 0 – a perfect resistor. For flat metal electrode the value is close to 1. The decrease in *α* indicates roughening of the surface upon phosphorylation. This is in agreement with our model and the AFM results, showing the reversible formation of pinholes upon phosphorylation of the monolayer.

### Biological implications

HDGF^27,46^ is a secreted protein^46,47^ that stimulates growth of several cell types including smooth muscle cells,^48^ endothelial cells^49^ and lung epithelial cells.^20,21,36,51^ HDGF was previously linked to lung cancer and specifically to poor clinical outcome. It was shown to cause tumorigenesis *in vivo*.^52^ Exogenously supplied HDGF was found to enhance the invasiveness of breast cancer.^47^ Inhibition of HDGF using antibodies showed anti-tumor activity.^50^ The observation that HDGF is over-phosphorylated in NSCLC tissues is in agreement with previous studies, which showed that the S165A mutation in HDGF prevented the secretion of HDGF. The S165D mutation, which introduces a negative charge as the phosphorylation does, caused secretion of HDGF.^46^ Therefore, the phosphorylation on HDGF S165 is a valid target for cancer therapy based on kinase inhibitors.

ERK1/2, also known as Mitogen Activated Protein Kinases 3/1 (MAPK3/1), are part of the MAPK/ERK pathway that communicates signals from the cell surface receptors to the nucleus.^2,5^ ERK1 and ERK2 share 83% amino acid identity, but despite their high similarity, they have distinct biological roles.^53–55^ They promote cell proliferation and metastasis.^2^ Over-activation of ERK1/2 is common in cancer, including NSCLC,^9,41,56^ prostate and breast cancers.^57,58^ Overexpression of HDGF causes activation of ERK1/2.^59^ Residues 8–17 on HDGF are a possible MAPK docking motif with 100% similarity to the consensus sequence.^46^ We show that HDGF S165 is a likely substrate of ERK2, and therefore inhibition of ERK2 could possibly inhibit the secretion of the phosphorylated HDGF, thus decreasing its tumorigenic activity.

## Conclusions

In this study we combined mapping of over-phosphorylated proteins in tumor tissues, quantification of the extent of over-phosphorylation that points to abnormal kinase activity in the tissue and the detection of a kinase that is likely to phosphorylate the substrate peptide sequence on the selected target protein. Using the model system of NSCLC, we demonstrated a sensitive integrated approach for targeted substrate–kinase pair selection based on differential phosphoproteomic profiling between cancer and adjacent normal tissues. This provides the advantage of individualized analysis as well as a generic platform to be applied to a specific type of cancer. The phosphorylating kinase can be predicted by bioinformatics tools and verified by phosphorylation *in vitro* and MS/MS sequencing to confirm the phosphorylation site. The developed label-free electrochemical assay then provides rapid and sensitive quantification of the substrate-dependent kinase activity without the requirement for specific antibodies or electrochemical labels. Therefore our methodology is general and could be applied for a panel of substrate–kinase biomarkers. Integrated approaches for the mapping of phosphorylation sites and prediction of their corresponding kinase have been previously suggested.^60^ Our method shows, however, a simpler implementation from the patient to the detection stage. It can be used for finding new drug targets, for mapping over-phosphorylation in specific cancer types and matching specific conditions for a specific overactive kinase, thus facilitating the design of specific kinase inhibitors as personalized drugs.

## Experimental

### Chemicals

Triethylammonium bicarbonate (TEABC), iron-(iii) chloride (FeCl_3_), formic acid, acetic acid, HPLC-grade acetonitrile (ACN) and all of the other chemical reagents were obtained from Sigma-Aldrich (St. Louis, MO, USA). The bicinchoninic acid assay (BCA) protein assay kit was obtained from Pierce (Rockford, IL, USA). Modified sequencing-grade trypsin was purchased from Promega (Madison, WI, USA). Ammonium persulfate (APS) and *N*,*N*,*N*′,*N*′-tetramethylenediamine (TEMED) were purchased from Amersham Pharmacia (Piscataway, NJ, USA). Polypropylene frits disk was purchased from Agilent (Wilmington, DE, USA). Ni-NTA silica resin was purchased from Qiagen (Hilden, Germany). SDB-XC Empore disks were obtained from 3 M (St. Paul, MN). Water was obtained from a Millipore Milli-Q system (Bedford, MA). Cell lysis buffer was obtained from Cell Signaling Technology, Inc.

### Protein extraction and digestion

We received the tissue from National Taiwan University Hospital and the approved IRB case number was 201104061RC (approved by National Taiwan University Hospital, IRB case number: 201104061RC). Frozen tissues were thawed rapidly at 37 °C, cut into small pieces, and washed by PBS to remove surface blood from tissues. The pre-cleaned tissues were homogenized in Triton cell lysis buffer containing 20 mM Tris–HCl (pH 7.5), 150 mM NaCl, 1 mM Na_2_EDTA, 1 mM EGTA, 1% Triton, 2.5 mM sodium pyrophosphate, 1 mM *b*-glycerophosphate, 1 mM Na_3_VO_4_ and 1 μg ml^–1^ leupeptin (cell signaling). By bead mill homogenizer (Precellys 24 Bead Mill Homogenizer, Bertin Technologies, MD), the pre-cleaned 50 mg tissue samples were put into one tube contain 10 beads in 200 μL lysis buffer. The tissues were crashed in 20 seconds at 5500 rpm under 4 °C. The protein concentration was determined by BCA protein assay kit.

The protein samples from tissue samples were subjected to gel-assisted digestion.^23^ The protein sample was fixed into a gel directly in the Eppendorf vial with acrylamide/bisacrylamide solution (40%, v/v, 29 : 1), 10% (w/v) ammonium persulfate, 100% *N*,*N*,*N*′,*N*′-tetramethylenediamine by a 14 : 5 : 0.7 : 0.3 ratio (v/v). The gel was cut into small pieces and washed several times with 25 mM TEABC containing 50% (v/v) ACN. The gel samples were further dehydrated with 100% ACN and then completely dried by vacuum centrifugation. Trypsin was then added into gel for proteolytic digestion (protein : trypsin = 50 : 1, g g^–1^) in 25 mM TEABC with incubation overnight at 37 °C. Digested peptides were extracted three times with 5% (v/v) FA in 50% (v/v) ACN for 30 min, dried completely by vacuum centrifugation at room temperature.

### Phosphopeptide purification by IMAC

Phosphopeptide purification was performed using an IMAC protocol.^24,25^ The in-house-constructed IMAC tip was capped at one end with a 20 μm polypropylene frits disk enclosed in a tip-end fitting. The tip was packed with 20 mg of Ni-NTA silica resin. First, Ni^2+^ ions were removed with 50 mM EDTA in 1 M NaCl. The tip was then activated with 100 mM FeCl_3_ and equilibrated with loading buffer (6% (v/v) acetic acid (AA) at pH 3.0) prior to sample loading. Tryptic peptides were reconstituted in loading buffer and loaded onto the IMAC tip. After successive washes with 6% (v/v) AA, 25% ACN, and 6% (v/v) AA, the bound peptides were eluted with 200 mM NH_4_H_2_PO_4_. The eluted peptides were desalted using reverse phase-Stage Tips(SDB-XC).^61^

### Mass spectrometry analysis

Purified phosphopeptides were reconstituted in buffer A (0.1% FA in H_2_O) and analyzed by liquid chromatography tandem mass spectrometry (LC-MS/MS). The TripleTOF 5600 system (AB SCIEX, Canada) equipped with a nanoACQUITY UPLC (Waters Corporation, Milford, MA, USA) was employed for nanoLC-MS/MS analysis. The samples were loaded and separated on a 15 cm column with 100 μm inner diameters, packed in-house with 3 μm C18 particles (Dr Maisch, Ammerbuch, Germany). The LC mobile phase system consists of water with 0.1% acetic acid (buffer A) and acetonitrile with 0.1% acetic acid (buffer B). The separation gradient of buffer B was up to 80% in 120 min with flow rate of 500 nL min^–1^ in nanoACQUITY UPLC system. The TripleTOF 5600 was fitted with a Nanospray III source. Data was acquired using an ion spray voltage of 2.5 kV, curtain gas of 20 PSI, nebulizer gas of 15 PSI, and an interface heater temperature of 150 °C. For information dependent acquisition (IDA), MS survey scan range was *m*/*z* 300–1500 acquired in 250 ms. The top 10 precursor ions were selected if exceeding a threshold of 100 counts per second (counts per s) in each MS survey scan, and subsequent 10 MS/MS scans were performed for 200 ms each. Collision energy setting of 40 ± 15 eV was applied to all precursor ions for collision-induced dissociation. To minimize repeated scan, dynamic exclusion was set for 6 s and then the precursor was refreshed off the exclusion list.

### Data processing, phosphoprotein identification and quantitation

Raw MS/MS data from the TripleTOF 5600 were transformed to mgf-files using the software AB SCIEX MS Data Converter (AB SCIEX, Canada). The mgf-files were searched using Mascot against the Swissprot database (Homo_sapiens) with the following settings: the fragment ion mass tolerance was set at 10 ppm, and the parent ion tolerance at 0.1 Da; for phosphopeptide search, phosphorylation (STY) and oxidation (M) were specified as variable modifications; for protein search, only oxidation (M) were specified as variable modifications. Peptides were considered identified when their Mascot Significance threshold is set as *p* < 0.05.

To evaluate the protein identification false discovery rate (FDR), we repeated the searches using identical search parameters and validation criteria against a randomized decoy database created by Mascot. For phosphorylation site determination, we reported the difference in Mascot scores of peptides with top two assignments on different phosphorylated sites (delta score).^62^ If the delta score is greater than 5, the top ranked phosphorylated site is considered confidently determined. For delta score less than 5, the phosphorylated site assignment is considered ambiguous.

The quantitative analysis of phosphopeptide was performed by IDEAL-Q.^26^ The raw data files from the TripleTOF 5600 were transformed to mzML format using the AB SCIEX MS Data Converter. The search results in MASCOT were exported in eXtensive Markup Language data (.XML) format. After data conversion, the confident peptide identification results (*p* < 0.05) from each LC-MS/MS run were loaded and merged to establish a global peptide information list (sequence, elution time and mass-to-charge). Alignment of elution time was then performed based on the peptide information list using linear regression in different LC-MS/MS runs followed by correction of aberrational chromatographic shift across fragmental elution-time domains. To increase correct assignment, the detected peptide peaks were validated by the SCI validation using the three criteria: (a) signal-to-noise (*S*/*N*) ratio > 3, (b) accurate charge state and (c) correct isotope pattern. To calculate relative peptide abundance, the tool performs reconstruction of extracted ion chromatography (XIC), and calculation of XIC area. The fold-change of a given phosphopeptide was calculated by the ratio of relative peptide abundance between different samples.

### *In vitro* phosphorylation of HDGF 160–174 *in vitro* kinase reaction

5 μL of 0.11 mM HDGF 160–174 solution (1 μg) were mixed with 5 μL of 2× kinase reaction buffer from Promega ADP Glo kinase assay (160 mM Tris–HCl, 80 mM MgCl_2_, 0.4 mg mL^–1^, 0.2 mM DTT), 5 μL of 250 μM ATP solution (Promega), and 4 μL of ERK2 as kinase (p42 MAP Kinase, New England Biolabs Inc.). The mixture was allowed to react for 4 hours at 30 °C under constant shaking. Afterwards, the reaction was stopped by cooling on ice and acidifying the solution with TFA (0.5% v/v final concentration). The mixture was desalted using reverse phase-Stage Tips (SDB-XC)^61^ and the phosphopeptides were enriched using a Fe-IMAC protocol previously described.^24,25^

### Electrochemical characterization of phosphorylation

#### Gold electrodes preparation

Polycrystalline bulk gold electrodes with a surface area of 4 mm^2^ were used for electrochemical measurements (CH instruments). These electrodes were hand-polished on micro-cloth pads (Buehler, Lake Bluff, IL) with de-agglomerated alumina suspension (Buehler) of decreasing particle size (1.0 and 0.05 μm) using a homemade polisher. After polishing the electrodes were sonicated in triple distilled water (TDW) for 15 min followed by additional sonication in EtOH solution (50% v/v) for 10 min. The washed gold electrodes were then cleaned with warm HNO_3_ (70%) for 20 min. The electrodes were then rinsed with TDW and cycled from –0.4 to 1.6 V in 0.5 M H_2_SO_4_ at 0.1 V s^–1^ until stable reproducible cyclic voltammograms were obtained representing the electrochemical reduction of an AuO monolayer.

#### Electrode surface modification

The peptide used in the monolayer experiments was HDGF 160–174 with an added C-terminal cysteine for attachment to the gold surface (CDLLEDSPKRPKEAEN) (Biomatik, Canada). Treated gold macroelectrodes were immersed into prepared HDGF 160–174 peptide solution (0.1 mM) and were kept for 16 hours at 25 °C. After removal, the electrodes were rinsed three times in TDW and dried under nitrogen gas stream. Electrochemical characterization was preformed directly after the electrodes were washed. The peptide solution was prepared by dissolving the peptide in weakly basic TDW.

#### Kinase catalysed phosphorylation

Peptide modified gold electrodes were reacted with Glutathione S-transferase (GST) – tagged ERK2 (New England Biolabs). Kinase mediated phosphorylation was performed by dripping of kinase solution on modified electrodes surfaces at 37 °C. Reaction medium (3 μM kinase in a final reaction volume of 40 μl) 80 mM Tris–HCl pH = 7.5, 40 mM MgCl_2_, 0.2 mg mL^–1^ BSA and 100 μM ATP. The reaction was initiated with the addition of the freshly prepared protein kinase solution and stopped by rinsing, after 30 min. Electrodes were washed multiple times in TDW, followed by a brief sonication in TDW.

Phosphorylation with CaMK2 was done with CaMK2 (New England Biolabs) in a reaction medium containing 3 μM kinase in a final reaction volume of 40 μl) including 80 mM Tris–HCl pH = 7.5, 40 mM MgCl_2_, 0.2 mg mL^–1^ BSA, 2.4 μM calmodulin, 4 μM CaCl_2_ and 100 μM ATP.

#### Phosphatase catalysed dephosphorylation

Hydrolysis of surface grafted phosphorylated peptides was performed in the presence of AP, 0.1 Units in a final reaction volume of 40 μl, for 30 minutes at 37 °C. 1 Unit AP was dissolved in a 1 ml of tris borate buffer, (pH 8.5), containing 10 mM MgCl_2_. (One unit (U) is defined as the amount of enzyme that can hydrolyze 1 μmol of phosphorylated peptide in a total reaction volume of 1 ml in 1 min at 37 °C).

#### Electrochemical characterizations

Electrochemical measurements were taken following each modification step. EIS measurements were performed using an Autolab PGSTAT12 digital potentiostat (EcoChemie BV, Utrecht, The Netherlands) connected to NOVA software. A conventional three-electrode cell was employed, with a peptide modified gold electrode as working electrode, and a standard Ag/AgCl reference electrode (Metrohm) with a KCl concentration of 3 M. The EIS measurements solution contained 5.0 mM K_3_[Fe(CN_6_)], 5.0 Mm K_4_[Fe(CN_6_)], and 0.1 M of KCl as supporting electrolyte. The spectra were recorded at a frequency range of 0.1 Hz–10 kHz with an amplitude of 10 mV, at the formal potential of the redox couple *vs.* Ag/AgCl electrode. The peptide adsorption was done over 15 hours. A three electrode cell, containing a clean gold electrode, was filled with a solution of 0.10 M phosphate buffer, pH = 8, that was also used as the supporting electrolyte, with added redox species: 5.0 mM K_3_Fe(CN_6_) and 5.0 mM K_4_Fe(CN_6_). The measurement was started, and after it reached a stable signal, a concentrated peptide solution was injected into the cell, to a final peptide concentration of 0.1 mM. Data points were measured every 25 s, at 100 Hz. The data was fit to an appropriate equivalent circuit using NOVA software.

### AFM characterizations

The substrates for AFM scans were freshly prepared annealed gold with atomically flat terraces.^35^ A HDGF 160–174 peptide layer was deposited by submerging the substrate immediately after annealing in peptide solution (0.1 nM) overnight. The samples were then washed with TDW and dried in a flow of nitrogen gas. AFM scans were performed with a Smart-AIST system (AIST-NT, SmartSPM™ 1000) using Olympus tips (OMCL-RC800PSA, Olympus Optical Co. Ltd) with cantilever force constant of 0.76 N m^–1^ and tip radius of 15 nm. To reduce surface damage, the scanning mode was true non-contact – verified by using tip-surface distance which was larger than the oscillation amplitude and with positive phase shift. Several macroscopically distant locations on each sample were scanned to eliminate “local effects” and at least five images of 1 μm × 1 μm were taken at each position. Scratches were done with 200 nN force. The scanning resolution was 1 pixel per nm. Image processing and analysis were done using WSxM 5.0 develop 7.0 software.^63^
