# Supplementary material for: Integrating proteomics with electrochemistry for identifying kinase biomarkers
Source: Chem Sci. 2015 May 22;6(8):4756–66. doi: 10.1039/c5sc00560d (PMC5667508; doi:10.1039/c5sc00560d)
Supplement: Supplementary file 1 [file SC-006-C5SC00560D-s001.pdf]

Electronic Supplementary Material (ESI) for Chemical Science.
This journal is © The Royal Society of Chemistry 2015

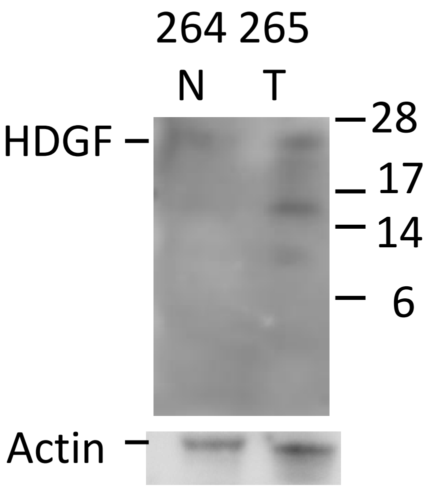

Supplementary Figure 1. Western blot validation of HDGF protein expression in tissue sample. N: normal tissue sample. T : tumor tissue sample.

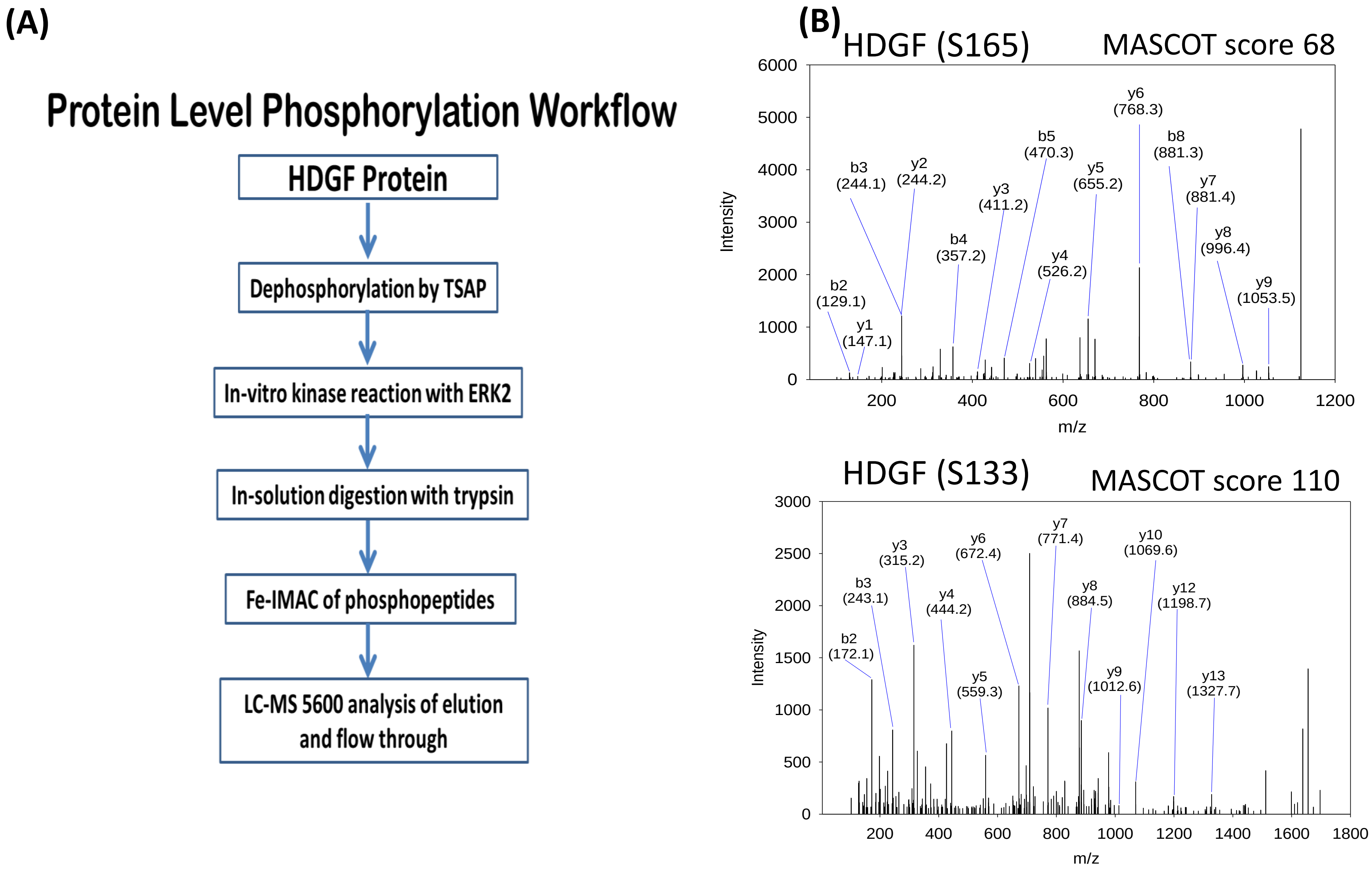

Supplementary Figure 2 (A) Workflow to perform in vitro kinase reaction for HDGF at protein level **(B)** MS/MS spectra of phosphopeptides S165 (AGDLLEDpSPK, m/z = 562.7) and S133 (GNAEGSpSDEEGKLVIDEPAK, m/z = 709.0)
